# Case report: Amplatzer septal occluder device migration into the descending thoracic aortic isthmus: percutaneous retrieval and redeployment

**DOI:** 10.3389/fcvm.2023.1269032

**Published:** 2023-10-13

**Authors:** Kun Xiang, Qi Ai, Lin He, Chengming Fan

**Affiliations:** ^1^ Department of Cardiovascular Surgery, the Second Xiangya Hospital, Central South University, Changsha, China; ^2^ Department of Cardiology, Shaoyang Central Hospital, Shaoyang, China

**Keywords:** atrial septal defect, percutaneous closure, amplatzer septal occluder device, occluder embolization, percutaneous retrieval

## Abstract

Percutaneous closure has emerged as the standard treatment for secundum-type atrial septal defects (ASDs). However, there is a rare but serious complication of occluder device migration and embolization to the heart chambers or distal vasculature during or shortly after implantation. Although this occurrence is extremely rare, it can have disastrous consequences. Fortunately, advancements in equipment and technology have facilitated the transition from surgical procedures to percutaneous techniques for removing embolized occluder devices. In this report, we present a case in which an Amplatzer septal occluder (ASO) device embolized to the descending thoracic aortic isthmus two days after implantation. The device was successfully retrieved using a percutaneous technique, and another ASO device was subsequently redeployed to the ASD. Regrettably, the patient experienced an intraoperative cardiac arrest. Despite prompt rescue efforts and recovery of vital signs, the patient still suffered postoperative sequelae. The main reason for occluder device migration in this case may have been the undersizing of the ASO device due to the operator's lack of caution.

## Introduction

Secundum-type atrial septal defects (ASDs) are among the most common congenital heart diseases, with an incidence of approximately 3.78 per 100,000 live births, accounting for 6%–10% of all congenital heart defects (1). Percutaneous closure of secundum-type ASDs using the Amplatzer septal occluder (ASO) device, which was approved by the American Food and Drug Administration (FDA) in 2001, has proven to be safe and effective in both pediatric and adult patients, becoming the standard treatment for this type of ASD (2–6). However, device embolization is a rare and severe complication (2, 7), with most cases being identified during the procedure or immediately after (2, 8). The incidence of device embolization ranges from 0.3% to 2.2%, regardless of the size of the occluder device, ASD size, or the physician's expertise (5, 7, 9, 10). Reported embolization sites include all four cardiac chambers, the aorta, and pulmonary arteries (2, 7). While embolization to the right-sided heart, including the pulmonary artery, is more common, embolization to the extracardiac aorta (aortic arch, descending aorta, and abdominal aorta) is extremely rare (2). It has been reported that although 50%–70% of displaced occluder devices have been successfully retrieved percutaneously (5, 7), 60% of cases that embolize to the extracardiac aorta result in severe complications and/or require open-heart surgical retrieval, with only 40% managed through percutaneous techniques (8, 11–14). In this report, we present a case in which an ASO device embolized to the descending thoracic aortic isthmus two days after implantation. The embolized occluder device was successfully retrieved percutaneously by an experienced physician, and a larger device was redeployed to the ASD. Unfortunately, the patient experienced an intraoperative cardiac arrest, and although the patient's vital signs were restored through timely rescue efforts, postoperative sequelae persisted.

## Case report

**Figure 1:**
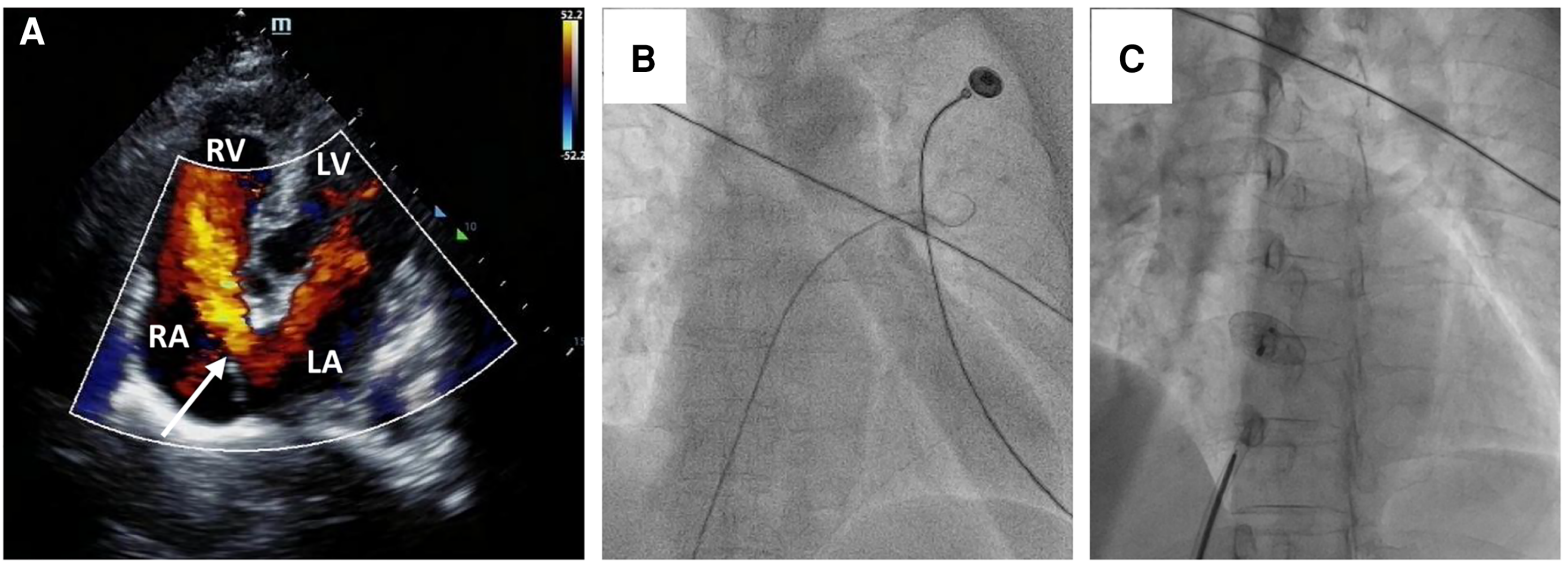
Secundum-type ASD finding and percutaneous secundum-type ASD closure. Transthoracic echocardiography revealed about 22 mm ASD (white arrow) with shunt flow from left atrium to right atrium (**A**) The intraoperative fluoroscopy showed that the guidewire passed through the inferior vena cava and the right atrium sequentially, and then through the defect site to the left atrium (**B**); a 24 mm ASO device was successfully deployed in the ASD (**C**).

**Figure 2:**
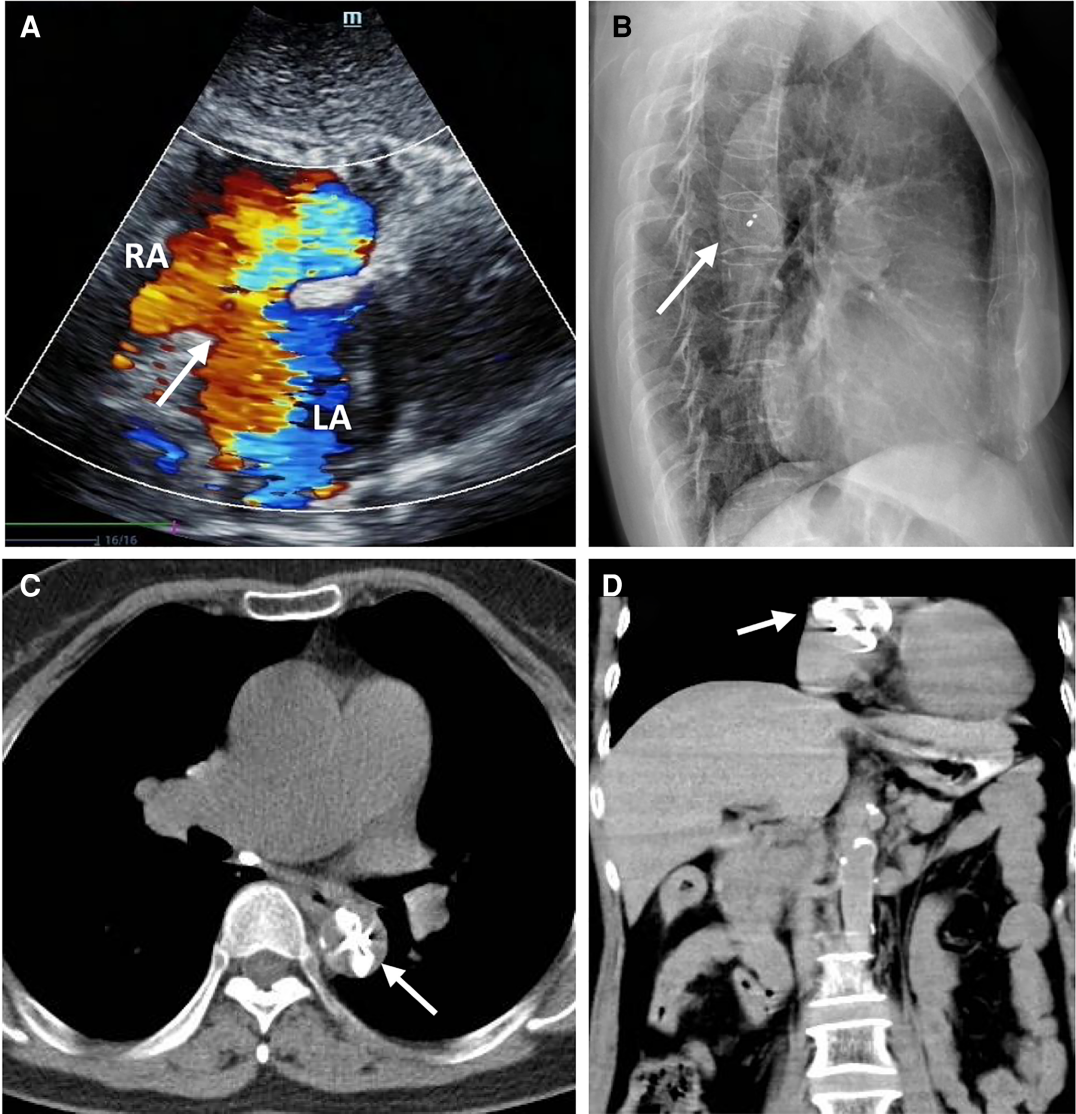
The ASO device embolization. Transthoracic echocardiography showing no occluder device and an interrupted echo (white arrow) in the middle of the atrial septum associated with a large number of left to right shunt flow (**A**); The lateral chest x-ray showed that the embolized occluder was located in the descending aorta (white arrow) (**B**); The chest CT showed that the embolized occluder device (white arrow) was located at the descending thoracic aortic isthmus (transverse view, **C**), (coronal view, **D**).

**Figure 3:**
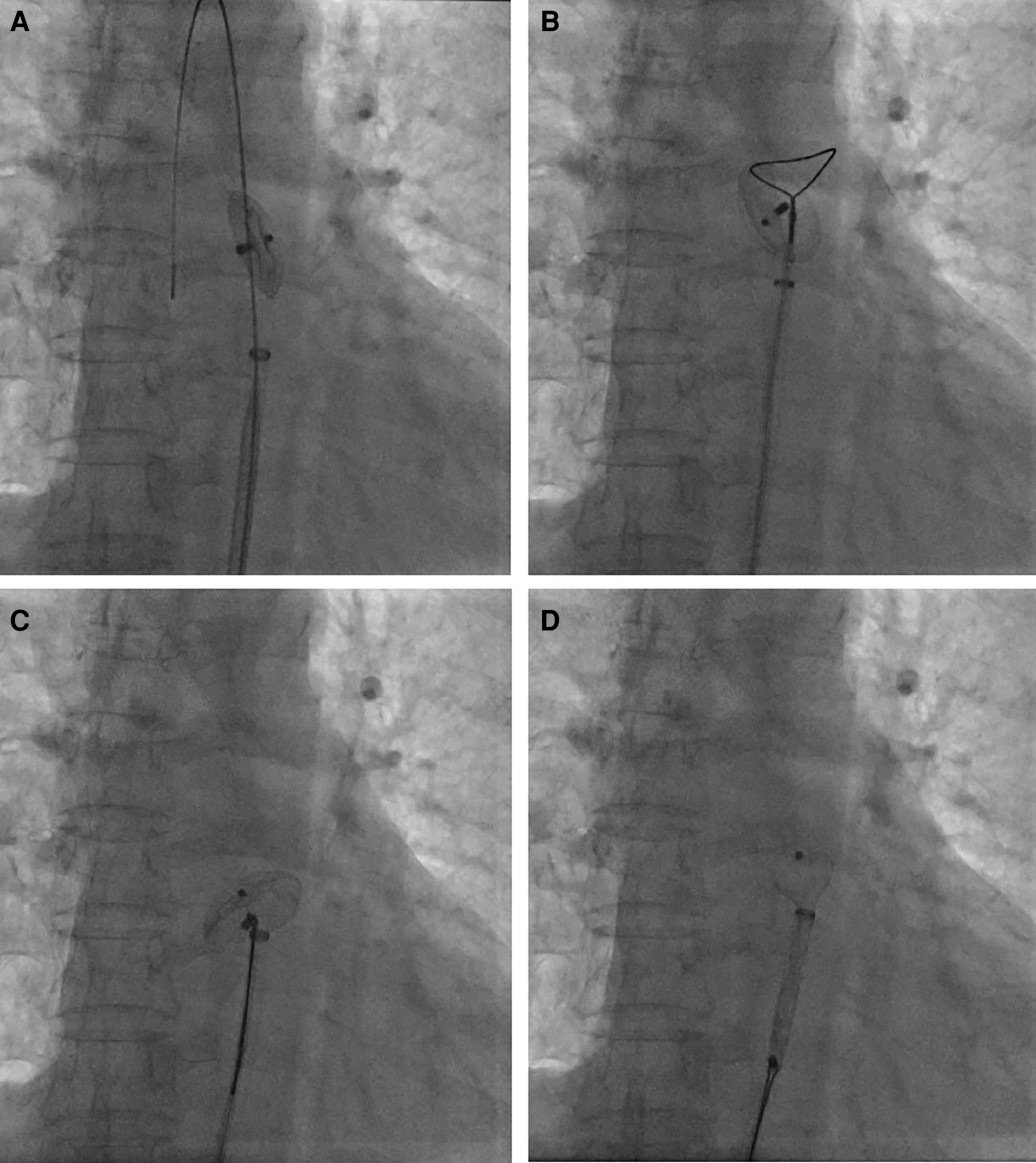
Percutaneous retrieval of the ASO device. Fluoroscopy revealed the embolized ASO device in the descending thoracic aortic isthmus (**A**); an Amplatz Goose Neck snare was advanced to the near occluder (**B**); after successful snaring of the screw on right atrial disc of ASO device (**C**), ASO device was successfully retrieved into the 12-French vascular sheath (**D**).

A 58-year-old asymptomatic woman residing in a remote countryside area was diagnosed with a secondary-type ASD during an evaluation for a heart murmur. Electrocardiography revealed a normal sinus rhythm with incomplete right bundle branch block. Local transthoracic echocardiography demonstrated a delicate and weak area measuring approximately 22 mm in the middle of the atrial septum, exhibiting movement synchronized with the cardiac cycle and abnormal left-to-right shunt flow, along with mild dilation of the right ventricle and atrium (Figure 1A). The physical examination revealed no contraindications for percutaneous closure. The intraoperative fluoroscopy confirmed the findings, as the guidewire sequentially passed through the inferior vena cava, right atrium, and defect site to the left atrium (Figure 1B). Given the delicate and weak area in the middle of the atrial septum, a 24 mm ASO device was successfully deployed under fluoroscopic guidance. The position of the occluder device was optimal, with no residual shunt observed on fluoroscopic examination after the final release (Figure 1C, arrow). However, two days after implantation, a heart murmur reemerged, and immediate transthoracic echocardiography revealed no occluder device in the middle of the atrial septum, accompanied by a significant left-to-right shunt flow (Figure 2A). Chest x-ray indicated that the occluder was located in the descending aorta (Figure 2B). The patient was then referred to our cardiac center. Computed tomography of the chest and abdomen confirmed the embolization of the occluder device to the descending thoracic aortic isthmus, with no evidence of vascular dissection, extravasation, or limb ischemia (Figures 2C,D). Since transthoracic echocardiography revealed no significant abnormalities in the anatomy and function of the mitral and aortic valves, and there were no significant hemodynamic changes, the patient underwent percutaneous retrieval of the embolized occluder device and subsequent redeployment under local anesthesia (Figure 3).

**Figure 4:**
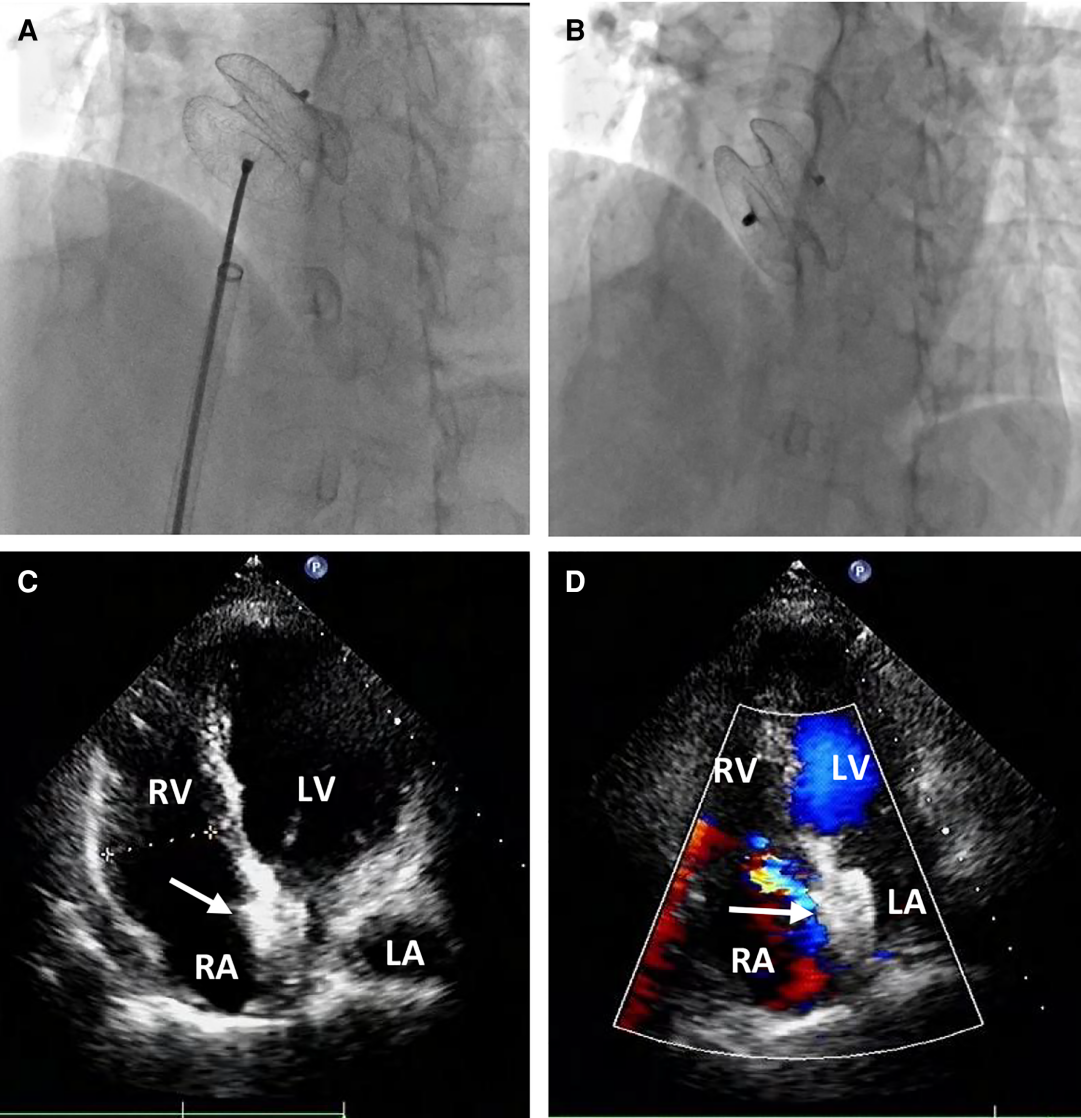
Percutaneous redeployment of the ASO device. Fluoroscopy revealed the 26 mm occluder was sent into the left atrium and the two sides of the occluder were successfully deployed on both sides of the defect (**A**); the position and shape of the redeployed occluder (white arrow) were found to be optimum, and no residual shunt (**B**) The occluder was located in the middle of the atrial septum (**C**) with no residual shunt (**D**) during the follow-up period.

Percutaneous retrieval and redeployment were performed via puncture of the right femoral artery and femoral vein, with insertion of 6F and 8F vascular sheaths, respectively. Chest fluoroscopy confirmed the embolization of the occluder device to the descending thoracic aortic isthmus (Figure 3A). Using a 12-French vascular sheath inserted through the right femoral artery, an Amplatz Goose Neck snare kit device was advanced to the embolized site (Figure 3B). The pedicle of the occluder was exposed by adjusting its position, allowing successful capture by the Amplatz Goose Neck snare under fluoroscopic guidance (Figure 3C). The displaced occluder was pulled into the vessel sheath (Figure 3D) and ultimately removed from the body through the puncture site in the right femoral artery. A 14-French vascular sheath was inserted at the right femoral vein puncture site and guided successively through the inferior vena cava, right chamber, left atrium, and left upper pulmonary vein. Subsequently, a 26 mm occluder was advanced into the left atrium using the long vascular sheath, and both sides of the occluder were successfully deployed on either side of the defect (Figure 4A). Chest fluoroscopy confirmed the optimal position and shape of the redeployed occluder, with no residual shunt (Figure 4B). The operator performed the “Minnesota wiggle” maneuver to ensure a secure position of the occluder device. However, during the procedure, the patient experienced an intraoperative cardiac arrest, which was promptly managed through cardiopulmonary resuscitation, leading to the recovery of vital signs. Subsequently, angiography of the aorta and pulmonary arteries revealed no evidence of dissection, extravasation, or residual shunt.

Following the procedure, the patient was hospitalized in the cardiac care unit for approximately nine days due to hypoxic-ischemic encephalopathy (HIE) and acute cerebral infarction. On the first postoperative day, the patient experienced intermittent convulsions under moderate intravenous sedation. Immediate head CT revealed significant swelling of the brain tissue in the right frontal, parietal, and occipital lobes, accompanied by shallow brain sulci and blurred boundaries between gray and white matter. The neurologist attributed these findings to HIE resulting from the cardiac arrest and initiated mild hypothermia therapy and cranial pressure reduction. On the third postoperative day, the patient no longer experienced convulsions but developed fatigue and reduced skin sensation in the left upper limb. A subsequent head CT scan revealed an acute and substantial area of cerebral infarction in the left cerebellar hemisphere, with a reduction in the swelling of the right frontal, parietal, and occipital lobes. The neurologists determined that thrombolytic therapy was unnecessary and prescribed medication to reduce cranial pressure and improve cerebral circulation. At discharge, on the ninth postoperative day, the patient exhibited sequelae of left upper limb dysfunction and was subsequently transferred to a local hospital for further treatment. During one month of clinical follow-up, the patient did not experience any cardiac symptoms or events, and transthoracic echocardiography confirmed the appropriate position of the occluder in the middle of the atrial septum, with no residual shunt (Figures 4C,D).

## Discussion

Percutaneous closure using the ASO device, approved by the FDA in 2001, has been established as a safe and effective treatment for secundum-type ASDs in both pediatric and adult patients (2–6). However, the displacement and embolization of the occluder device remain significant concerns. The occluder device has been reported to displace to all four cardiac chambers and the great vessels, including the pulmonary artery and aorta (8). Various factors contribute to occluder device embolization, such as inappropriate sizing, inaccurate deployment, inadequate operator experience, and tearing of the ASD during occluder device manipulation and friction (2, 7–10). In addition, a retrospective study revealed that the pulmonary to systemic blood flow ratio (Qp/Qs) >3.13 and eroded or floppy interatrial septum (IAS) or aneurysm formation post-implantation might be predictors of ASO dislodgement in adults and children (15). In our case, the migration of the occluder device may be attributed to the presence of a large, delicate, and weak area in the middle of the atrial septum. The delicate and weak atrial septum is prone to tearing when initially placing a 24 mm occluder, resulting in insufficient residual edge to secure the device. Ultimately, a 26 mm occluder successfully closed the ASD. Typically, operators are hesitant to use a larger occluder device due to concerns about excessive pressure on the aorta, valves, vessel walls, and anterior left atrial wall reflection. Furthermore, it has been reported that placing an occluder device in such a position increases the risk of cardiac erosion (7, 16–18). However, considering the presence of a large, delicate, and weak area in the middle of the atrial septum, operators should conduct comprehensive examinations, such as TEE, cardiac CT or MRI, to thoroughly understand the surrounding anatomical structures before proceeding with the procedure. Failure to do so may lead to catastrophic complications, including migration and embolization, due to inappropriate occluder device sizing or manipulation. In summary, undersizing the occluder device or inadequate and floppy rims due to the operator's lack of caution are the primary reasons for occluder embolization.

Embolization sites have been reported in all four cardiac chambers, the aorta, and the pulmonary arteries (2, 7). Regardless of whether embolization occurs in the right or left heart systems, patients face potential risks when the occluder device obstructs blood flow (10, 19, 20). Although successful percutaneous retrieval has been achieved in 50%–70% of displaced occluder devices (5, 7), embolization to the extracardiac aorta has been associated with severe complications and/or the need for open-heart surgical retrieval in 60% of cases, with only 40% managed through percutaneous techniques (8). One of the key advantages of the ASO device is its retrievability before final release, allowing for percutaneous removal of dislodged and embolized devices (4). Percutaneous foreign body removal is widely accepted as a technique to remove displaced devices from the vasculature, minimizing the need for more invasive surgical procedures and reducing costs (1, 21). Various percutaneous devices and techniques have been developed, including gooseneck snares, tulip-shaped snares, large sheaths, pigtail catheters, Dormia baskets, alligator clamps, and bioptomes (1, 9). In our case, we initially planned to use an Amplatz Goose Neck snare to capture the pedicle on the right side of the occluder and pull it back into the sheath. However, the angulation of the embolized occluder within the aorta presented a challenge in capturing its pedicle. Typically, the operator would rotate the occluder to expose the pedicle using an endocardial biopsy forceps, facilitating snare capture.

Regrettably, in our case, the patient experienced intraoperative cardiac arrest following the procedure, which was successfully managed through cardiopulmonary resuscitation, leading to the restoration of vital signs. First of all, cardiac arrest occurred after the occluder device was fully deployed, and when the operator performed wiggle maneuver, there is almost normal ECG sign. Therefore, we believed that wriggling the device to make sure of the deployment is unlikely to produce cardiac arrest. The primary cause of the occluder dislodgement is a torn bit of the floppy interatrial septum. Secondly, the floppy interatrial septum and conduction bundles may be damaged by redeployed occluder device, which could induce cardiac arrest, and this is one of the reason why operators are often reluctant to deploy larger occluder devices. Finally, coronary embolism is also possible. In terms of preoperative coagulation function and transthoracic echocardiography examination, we did not find evidence of thrombosis, and the endovascular operation was carried out after the intraoperative routine heparinization and the ACT reached the standard. However, we believe that the possible cause of cardiac arrest was coronary embolism (air embolism). If the bubbles is not completely removed when the occluder device was assembled into the sheath, the bubbles will then be pushed to the left atrium, leading to coronary embolism. Further evidences showed that serum troponin increased approximately 143-fold on the second postoperative day. Usually, a cardiac arrest lasting for 1 min does not trigger such a high level of serum troponin. Therefore, we believed that the myocardial infarction was caused by the coronary embolism. All in all, this case serves as a valuable lesson for reflection and learning.

## Conclusion

Occluder embolization is a rare and severe complication that can occur during percutaneous closure of ASDs. We presented a case in which an ASO device embolized to the descending thoracic aortic isthmus two days after implantation. The embolized occluder device was successfully retrieved using a percutaneous technique, and another device was redeployed to close the ASD. The primary reason for occluder embolization in this case was the undersizing of the ASO device due to the operator's lack of caution.

## Data Availability

The raw data supporting the conclusions of this article will be made available by the authors, without undue reservation.
